# Latin American adults who regularly use macrodoses of psychedelics: a cross-sectional study

**DOI:** 10.1038/s41598-024-74590-3

**Published:** 2024-10-13

**Authors:** Oscar Véliz-García, Marcos Domic-Siede

**Affiliations:** 1 Escuela de Psicología, Universidad Católica del Norte, Antofagasta, Chile; 2 Laboratorio de Neurociencia Cognitiva UCN, Antofagasta, Chile; 3 Núcleo de Investigación en Neurociencia Afectiva, Cognitiva y Neuropsicología, UCN, Antofagasta, Chile

**Keywords:** Psychedelics, Macrodoses, Well-being, Consumption practices, Psilocybin, Human behaviour, Epidemiology

## Abstract

Psychedelics have a complex history marked by traditional use among indigenous cultures, early scientific interest, and subsequent prohibition. Despite their classification as controlled substances, recent decades have witnessed a resurgence of research into their therapeutic potential for various mental health conditions. However, most studies have focused on controlled clinical settings, leaving a significant gap in understanding how these substances are used in naturalistic contexts, particularly in Latin America. This study investigates the regular use of macrodoses of psychedelics among Latin American adults. We aimed to characterize the sociodemographic profiles, consumption practices, and subjective effects experienced by individuals who use psychedelics regularly. Data were collected via an online survey from 4,270 participants across several Latin American countries. Results indicated a diverse user base with varied motivations, predominantly psychological and spiritual well-being. The most frequently used substance was psilocybin mushrooms, with significant associations found between demographic variables and specific psychedelics used. The study provides new insights into the naturalistic use of psychedelics in Latin America, highlighting the need for informed, safe, and legal use frameworks.

**Supplementary Information:**

The online version contains supplementary material available at 10.1038/s41598-024-74590-3.

## Introduction

According to the United Nations World Drug Report 2023, an estimated 296 million people used drugs in 2021^1^. In this context, psychedelics are often associated with recreational and illicit use. However, they have a long history of safe use among the indigenous cultures of the Americas^2^ and Eurasia^3^.

The term ‘psychedelic’ is derived from the words ‘psyche’ (mind) and ‘delein’ (to make manifest), referring to the mind’s capacity for manifestation, which can bring useful and beneficial properties^4^. It is used to describe substances that enable the conscious manifestation of the unconscious, allowing the perception of emotions and experiences in non-ordinary states of consciousness^5^. Another term used to define these substances is “entheogens”, derived from the Greek ‘entheos’ (God) and ‘genos’ (generate), which refers to substances that generates the divine or God-like experiences in those who consume them, make it a synonym in the literature^4^. These substances generate significant transformations in perception, mood and thought, with minimal effects in orientation and memory^5^.

The interest of allopathic medicine in these substances arose with the discovery of Lysergic Acid Diethylamide (LSD) by Albert Hoffman in 1943 and its therapeutic potential for mental health^6^. The recreational use of these substances outside the experimental and clinical context, along with the political revolution of the countercultural movement of the 1960s, led the United States government to declare a federal ban on the use of psychedelics in 1968. This ban was ratified globally by the United Nations Convention on Narcotics in 1971^6^.

Despite these restrictions, research with psychedelic substances has continued over the past three decades^7^. Griffiths’ 2006 study is often considered the start of the psychedelic renaissance^6,7^ which was further advanced by Carhart-Harris and Nutt’s 2012 neuroimaging studies^8^.

Additionally, in recent years, several scientific reports have demonstrated the therapeutic potential of psychedelics to address a variety of mental health diagnoses. For example, psilocybin has shown promise for treating depression and anxiety^9–11^, 5-MeO-DMT and MDMA for post-traumatic stress disorder^12–15^, psilocybin for alcohol dependence^16^, and ibogaine for detoxification in opioid and cocaine abusers^17^.

As mentioned above, empirical studies in this decade have focused on the therapeutic potential of these substances in mental health and their neurodynamic correlates. However, we have limited knowledge of how psychedelics are used in naturalistic settings^18^. While there is existing data on psychedelic use in naturalistic and recreational settings in many countries and with some compounds (e.g., ayahuasca)^19^, there remains a gap in research characterizing the use of these substances and the people who use them in the Latin American context.

To address this gap, this study aims to explore three primary research questions. First, it seeks to identify the sociodemographic profiles of Latin American adults who regularly consume macrodoses of psychedelics. Second, it examines the consumption practices and variables associated with the regular use of psychedelics in Latin America. Third, it investigates the significant associations between sociodemographic variables, consumption variables, and specific psychedelics used, hypothesizing associations between variables such as gender, occupation, education, religion, context, company, and the types of psychedelics consumed. By addressing these questions, the study aims to provide a comprehensive understanding of the naturalistic use of psychedelics in Latin America, which can inform the development of safe and legal use frameworks.

## Methods

The study was approved by the Scientific Ethics Committee of the Universidad Católica del Norte (resolution n° 013/2023) and adhered to the principles of the Declaration of Helsinki and Singapore. Electronic informed consent was obtained from all participants before they responded to the survey.

The study employed an exploratory and cross-sectional design, focusing on adults who regularly use psychedelics. In the absence of a single clear definition of regular use, this criterion was operationalized as people who have been using psychedelics for at least three years with a frequency of at least four times per year (once per quarter).

The inclusion criteria required participants to be 18 years of age or older, able to read and understand Spanish, and meet the operationalization of habitual consumption. To encourage participation, the survey did not collect any personal identification data (e.g., name, national identification number, email). Given this design feature, the inclusion criteria could not be verified beyond the information provided by the participants themselves.

Data were collected between April 2023 and March 2024 from self-selected participants recruited through web-based announcements on social media services (Instagram and Facebook), advertisements, and interviews published in digital information channels of broadcasters, universities, and Non-Governmental Organizations related to psychedelics. Before starting the survey, participants viewed the following text: “*Have you consumed any psychedelic substances in your lifetime? By psychedelics we refer to products of organic origin (e.g. Psilocybin mushrooms*,* Ayahuasca*,* Peyote*,* etc.) or of synthetic origin (e.g. MDMA*,* LSD*,* 2 C-B*,* Ketamine. etc.) whose consumption has diverse effects on brain functioning*,* causing changes in perception*,* cognition*,* behavior*,* and emotions. If you have consumed any psychedelics*,* one or more times*,* are 18 years old or older*,* and live in a Latin American country*,* you can participate in this study.”*

The required sample size was determined using G*Power v3 for a chi-square test, as our analysis focused on examining associations between sociodemographic variables (independent), consumption variables (independent), and types of psychedelics used (dependent). The parameters were set for a large effect size (effect size w = 0.1), a significance level (α) of 0.01, a power (1-β) of 0.99, and degrees of freedom (df) of 5, resulting in a required sample size of 3,355 participants.

The online survey was developed by the research team and pilot-tested with a group of 50 people to assess linguistic comprehension and the correct functioning of the digital platform. It was subsequently reviewed by a group of subject matter experts composed of clinicians and psychologists. The survey considered three dimensions related to sociodemographic information, information on the first consumption experience, and information on regular use. For this publication, data from the first and last dimensions described above were considered. We decided to include only the regular consumption experience in this report, as the first consumption experience represents a complex phenomenon, and we wanted to address each process separately in greater depth.

To ensure that the participants’ answers were the result of thoughtful responses and not random selections, an attentional control question was included every five questions (e.g., “In this question, we ask you to mark the triangle option,” “In this question, select the option that represents the result of 3 + 3”).

One of the variables explored in the regular use of psychedelics is the ‘purpose of use’, which was defined as follows: *recreational purpose* (the search for pleasure, fun, and/or curiosity); *medicinal purpose* (the search to address a symptom and/or clinical condition of physical or psychological health); *spiritual purpose* (the search for connection with a higher and/or transpersonal dimension). The purpose of *Psychedelic-Assisted Psychotherapy* (PAP) consists of the use of psychedelic substances as part of the psychotherapy process. Additionally, the regular use dimension included the variable ‘frequency of consumption,’ operationalized as follows: *daily* (every day of the week); *weekly* (every week, but not every day of the week); *monthly* (only some days of the month); and *annually* (at least four times a year, but not every month).

For this report, we included only the psychedelic substances that emerged from the participants’ responses, based on the postulates of citizen science^20^, resulting in the inclusion of Psilocybin Mushrooms, Ayahuasca, San Pedro cactus, Peyote, Floripondium, Ibogaine, Salvia Divinorum, Anandenanthera Colubrina, DMT-Tepezcohuite, MDMA, LSD, Synthetic DMT, 2 C-B, and Ketamine (for additional information on scientific names, street names, native names, and psychoactive compounds of these substances, see supplementary table A).

### Statistical analyses

We used descriptive statistics and the chi-square (*X*²) statistic to compare demographic and regular use variables. All *X²* significance tests were performed at the *p* < 0.01 level using SPSS v29 Software. To address the multiple comparison problem in our analysis using multiple chi-square tests, all data were corrected using the False Discovery Rate (FDR) method^21^.

## Results

### Demographics

The online survey was completed by 4,270 participants from various Latin American countries, with the largest representation from Chile (53%, *n* = 2,263), followed by Colombia (16%, *n* = 683), Peru (10%, *n* = 427), Argentina (7%, *n* = 299), Bolivia (6%, *n* = 256), Ecuador (4%, *n* = 171), and Uruguay (4%, *n* = 171). The mean age of the participants was 31.9 years (SD = 8.4). Table 1 presents the distribution of participants according to sociodemographic variables.

**Table 1:** Distribution of participants according to sociodemographic variables.

Gender	
Female	56% (2391)
Male	39.6% (1691)
Non-binary	4.4% (188)
Age	
18–29	45.2% (1930)
30–64	54.6% (2331)
65+	0.2% (9)
Sexual orientation	
Heterosexual	72.1% (3079)
2SLGBTQIA+	27.9% (1191)
Sex-affective relationship	
Monogamous	56% (2391)
Polygamous	0.9% (38)
Open relationship	6.1% (260)
Don’t have a partner	37% (1580)
Occupation/Employment	
Out of Work	5.2% (222)
Housework	1.2% (51)
Student	16.2% (692)
Dependent Worker	34.7% (1482)
Self-employed Worker	21.5% (918)
Politician	0.2% (9)
Psychedelic-Assisted Psychotherapy Practitioner or Psychedelic Experiences Facilitator	6.1% (260)
Dependent and Self-employed Worker	6.3% (269)
Student and Dependent Worker	7.7% (329)
Student, Dependent and Self-employed Worker	0.7% (30)
Reteree	0.2% (9)
Personal monthly income (in USD)	
$0 to $698	49.6% (2118)
$699 to $1423	31.4% (1341)
$1424 to $3583	15.7% (670)
Above $3584	3.3% (141)
Education	
0–8 years	0% (0)
9–12 years	7.2% (333)
13–17 years	39.9% (1678)
Above 18 years	52.9% (2259)
Religion/Creed	
Catholic	6.6% (282)
Buddhist	2.6% (111)
Evangelical	1.4% (60)
Christian	1.4% (60)
Agnostic	1.2% (51)
Gnostic	0.5% (21)
Hare Krishna	0.2% (9)
Belief in something higher	3.7% (158)
Shamanism or Ancestral Beliefs	1.9% (81)
God/Jesus	0.7% (30)
Nothing (no religion or creed)	79.9% (3412)

### Regular psychedelic use

The mean age at first psychedelic use was 24.2 years (SD = 7.6), with a minimum age of 12 years and a maximum age of 67 years. On average, participants had consumed three different psychedelics during their lifetime (SD = 1.85). Table 2 shows the distribution of the number of substances consumed, as well as the distribution of each substance.

**Table 2:** Distribution of substances and the quantity consumed.

Number of substances consumed	
Just 1	33% (1409)
2	20.1% (858)
3	17.8% (760)
4	12.2% (521)
5	8.4% (359)
6	4% (171)
7	2.1% (90)
8	1.4% (60)
9	0.2% (9)
10 +	0.7% (30)
Substance distribution	
Psilocybin mushrooms	88.8% (3792)
MDMA	48.2% (2058)
LSD	40.3% (1721)
2 C-B	15.2% (649)
San Pedro cactus	16.4% (700)
Ketamine	15.2% (649)
Synthetic DMT	13.8% (589)
Ayahuasca	12.2% (521)
Salvia Divinorum	4.7% (201)
Peyote	4.2% (179)
Floripondium	3.3% (141)
Ibogaine	1.4% (60)
DMT-Tepezcohuite	1.4% (60)
Anandenanthera Colubrina	0.5% (21)

We identified 12 reasons why participants continued to use psychedelics regularly (Table 3). The primary reason was ‘The psychological well-being experienced during use’ (65.3%, *n* = 2,788). Additionally, 78.8% (*n* = 3,360) of participants described two or more reasons for regular consumption.

**Table 3:** Reasons for using psychedelics on a regular basis.

Reasons for continued use	
Psychological well-being experienced during use	65.3% (2788)
Spiritual well-being experienced during use	59% (2519)
The feeling of calm that lingers after use	50.6% (2161)
Curiosity to explore what else can be experienced under the effects of the substance	47.3% (2020)
The published scientific reports on the benefits of these substances	38.9% (1657)
The reduction or elimination of the psychological symptoms experienced prior to consumption	24.4% (1042)
The call of the substance itself, although I don’t really know how to explain it	18% (769)
The invitation of people close to me who regularly consume	13.6% (581)
The loss of fear of death	7% (299)
The suggestion of a professional, as part of treatment	5.9% (252)
The suggestion of a facilitator (e.g., Shaman, Paje, Taita, Machi, Medicine Man, etc.)	5.2% (222)
To continue the path of self-knowledge and/or the expansion of my consciousness	3.3% (141)
I had a bad trip the first time and wanted to have a better experience	2.8% (120)
To remain in contact with the whole, with the unity, with the divine, with the mystery	1.6% (68)
To continue to access information that appears in this modified state of consciousness	1.2% (51)
To seek inspiration and/or enhance my creativity	0.2% (9)

Regarding the purposes for which psychedelics are consumed, 67.9% (*n* = 2,899) reported using them for recreational purposes, 32.3% (*n* = 1,379) for medicinal purposes, 61.8% (*n* = 2,639) for spiritual purposes, and 6.8% (*n* = 290) for PAP purposes. Furthermore, 52.5% (*n* = 2,242) of the participants stated more than one purpose for their consumption.

Annual consumption frequency was the most common (64.9%, *n* = 2,771), followed by monthly (20.6%, *n* = 880), weekly (11%, *n* = 470), and daily (3.5%, *n* = 149).

The contexts of consumption included participants’ own homes (65.1%, *n* = 2,780), outdoors (64.4%, *n* = 2,750), acquaintances’ homes (39.3%, *n* = 1,678), strangers’ homes (4.9%, *n* = 209), educational institutions (3.5%, *n* = 149), parties (30.4%, *n* = 1,298), ceremonies or retreats (20.4%, *n* = 871), PAP sessions (2.3%, *n* = 98), and workplaces (2.1%, *n* = 90).

Regarding the company during consumption, the most frequent was with friends (72.4%, *n* = 3,091). Other percentages were distributed as follows: alone (49.2%, *n* = 2,101), with a partner (41.9%, *n* = 1,789), with a facilitator or professional (16.9%, *n* = 722), with family members (15.9%, *n* = 679), with strangers (7.7%, *n* = 329), and with a dealer (4%, *n* = 171).

The most frequent source of substance acquisition was a dealer (63%, *n* = 2,690), followed by friends (58.1%, *n* = 2,481), home-growing (22%, *n* = 939), facilitators or professionals (16.6%, *n* = 709), partners (7.7%, *n* = 329), purchase through an application (7.7%, *n* = 329), and family members (5.2%, *n* = 222). Most participants knew the amount of substance they consumed (81.5%, *n* = 3,480), while 18.5% (*n* = 790) did not. Participants spent an average of USD $135 (SD = 425.1) per year on psychedelics.

The subjective changes experienced due to regular use could be grouped into two categories: positive and negative. The most frequently reported positive changes were “increased capacity for self-observation” (67.7%, *n* = 2,891), “improved mood” (61.6%, *n* = 2,630), and “personal growth” (55.7%, *n* = 2,378). The most frequent negative changes were “increased questioning of my work, relationships, and/or habits with discomfort” (11.7%, *n* = 500), “decreased memory capacity” (10.1%, *n* = 431), and “increased difficulty in concentrating on work and/or daily activities” (8.7%, *n* = 371). Table 4 lists all the subjective changes reported, showing that positive changes were generally much more frequent than negative ones.

**Table 4:** Subjective changes reported.

Subjective changes reported	
Increased capacity for self-observation (e.g., more attentive to what I feel, think and/or do)	67.7% (2891)
Improved mood	61.6% (2630)
Personal growth	55.7% (2378)
Heightened sensitivity (e.g., I feel more connected to my emotions and/or those of other people)	54.8% (2340)
Decrease in anxiety and/or distress	53.4% (2280)
Spiritual growth	53.2% (2272)
Increased creativity	43.6% (1862)
Increased problem-solving skills	40% (1708)
Positive changes in my eating, hygiene and/or self-care habits	39.1% (1670)
General reduction of fears	36.5% (1559)
Physical well-being (e.g., decrease of ailments that used to be frequent)	34% (1452)
Changes in my recreational interests (e.g., music, sports, movies, literature, etc.)	29.5% (1260)
Increased questioning of my work, relationships and/or habits, without discomfort	23.9% (1020)
Distancing from people with whom I used to share frequently, without discomfort	22.5% (961)
Decreased fear of dying and/or of losing something/someone	21.5% (918)
Some degree of discomfort when sharing with groups of people (known or unknown),	16.9% (722)
Increased questioning of my work, relationships and/or habits with discomfort	11.7% (500)
Decreased memory capacity	10.1% (431)
Increased difficulty in concentrating on work and/or day-to-day activities	8.7% (371)
No significant change, everything remains as usual	6.3% (269)
Worse mood	4.7% (201)
Physical discomfort (e.g., frequent onset of post-consumption discomfort)	4% (171)
Negative changes in my eating, hygiene and/or self-care habits	3.5% (149)
Decreased sensitivity (e.g., I feel less connected to my emotions and/or those of other people)	1.6% (68)

### Regular psychedelic use and demographics

 The results showed significant relationships between sociodemographic variables and regular psychedelic use.

#### Gender

 Female gender was associated with the regular use of LSD (χ^2^ = 54.36, *p* < 0.01) and Ketamine (χ^2^ = 41.28, *p* < 0.01), while male gender was associated with the regular use of Synthetic DMT (χ^2^ = 34.81, *p* < 0.01), LSD (χ^2^ = 55.44, *p* < 0.01), and Ketamine (χ^2^ = 38.01, *p* < 0.01).

#### Occupation

 Being a student was associated with the regular use of Psilocybin mushrooms (χ^2^ = 44.80, *p* < 0.01) and LSD (χ^2^ = 35.97, *p* < 0.01), while being a psychedelic-experienced facilitator was associated with the consumption of Ayahuasca (χ^2^ = 47.88, *p* < 0.01) and DMT-Tepezcohuite (χ^2^ = 37.89, *p* < 0.01).

#### Education

 Psilocybin mushroom consumption was associated with groups having 13–17 years of education (χ^2^ = 36.47, *p* < 0.01) and more than 18 years of education (χ^2^ = 40.20, *p* < 0.01).

#### Religion or creed

 Not belonging to any religion or not professing any creed was associated with the regular use of Psilocybin mushrooms (χ^2^ = 50.13, *p* < 0.01), Ayahuasca (χ^2^ = 47.71, *p* < 0.01), LSD (χ^2^ = 47.69, *p* < 0.01), and 2 C-B (χ^2^ = 45.38, *p* < 0.01), while shamanism/ancestral beliefs was associated with the use of San Pedro cactus (χ^2^ = 46.71, *p* < 0.01), Peyote (χ^2^ = 38.72, *p* < 0.01), and Ayahuasca (χ^2^ = 49.30, *p* < 0.01). Belief in a higher power was associated with the regular use of San Pedro cactus (χ^2^ = 45.40, *p* < 0.01), Peyote (χ^2^ = 48.69, *p* < 0.01), and Ayahuasca (χ^2^ = 65.49, *p* < 0.01).

#### Income

 A monthly income below USD $648 was associated with the regular use of Psilocybin mushrooms (χ^2^ = 33.90, *p* < 0.01) and 2 C-B (χ^2^ = 35.53, *p* < 0.01), while a monthly income above USD $3,584 was associated with the use of Ketamine (χ^2^ = 34.70, *p* < 0.01), MDMA (χ^2^ = 35.83, *p* < 0.01), and Ibogaine (χ^2^ = 47.33, *p* < 0.01).

### Regular psychedelic use and consumption variables

 We also observed significant relationships between the substance regularly consumed and certain consumption variables.

#### Consumption context

 Consumption in one’s own house was significantly associated with the use of Psilocybin mushrooms (67.5%, χ^2^ = 38.41, *p* < 0.01). Outdoor settings were linked to the use of Psilocybin mushrooms (60%, χ^2^ = 44.53, *p* < 0.01), MDMA (35.1%, χ^2^ = 37.43, *p* < 0.01), LSD (29%, χ^2^ = 42.28, *p* < 0.01), San Pedro cactus (12.6%, χ^2^ = 35.92, *p* < 0.01), and Synthetic DMT (11.5%, χ^2^ = 40.38, *p* < 0.01). Consumption at an acquaintance’s house was related to LSD (24.8%, χ^2^ = 44.46, *p* < 0.01), MDMA (20.4%, χ^2^ = 45.24, *p* < 0.01), San Pedro cactus (9.4%, χ^2^ = 41.11, *p* < 0.01), 2 C-B (9.4%, χ^2^ = 45.82, *p* < 0.01), and Synthetic DMT (7.5%, χ^2^ = 36.36, *p* < 0.01).

The party context was significantly associated with MDMA (20.6%, χ^2^ = 88.38, *p* < 0.01), LSD (17.8%, χ^2^ = 37.81, *p* < 0.01), 2 C-B (9.8%, χ^2^ = 68.55, *p* < 0.01), and Ketamine (9.6%, χ^2^ = 68.55, *p* < 0.01). Ceremony contexts were linked to Psilocybin mushrooms (19.7%, χ^2^ = 36.65, *p* < 0.01), Ayahuasca (19.4%, χ^2^ = 146.70, *p* < 0.01), Peyote (13.3%, χ^2^ = 68.16, *p* < 0.01), San Pedro cactus (13.3%, χ^2^ = 123.12, *p* < 0.01), DMT-Tepezcohuite (11.4%, χ^2^ = 63.78, *p* < 0.01), Anadenanthera Colubrina (5.8%, χ^2^ = 37.85, *p* < 0.01), and Synthetic DMT (5.6%, χ^2^ = 47.39, *p* < 0.01). Educational settings were significantly associated only with 2 C-B (1.9%, χ^2^ = 47.49, *p* < 0.01).

#### Consumption purpose

 We observed a significant relationship between consumption for recreational purposes and consumption for spiritual purposes (χ^2^ = 76.46, *p* < 0.01).

#### Consumer company

 Consuming alone was significantly related to Psilocybin mushrooms (44.7%, χ^2^ = 45.22, *p* < 0.01), San Pedro cactus (12.3%, χ^2^ = 36.26, *p* < 0.01), and Salvia Divinorum (3.7%, χ^2^ = 37.97, *p* < 0.01). Consuming with friends was associated with LSD (40.5%, χ^2^ = 56.85, *p* < 0.01), MDMA (34.7%, χ^2^ = 56.95, *p* < 0.01), Ketamine (13.8%, χ^2^ = 42.98, *p* < 0.01), 2 C-B (14.3%, χ^2^ = 47.69, *p* < 0.01), and Salvia Divinorum (2.3%, χ^2^ = 35.24, *p* < 0.01). Consuming with a partner was associated with Psilocybin mushrooms (40%, χ^2^ = 44.16, *p* < 0.01), LSD (23.2%, χ^2^ = 36.15, *p* < 0.01), MDMA (20.1%, χ^2^ = 37.72, *p* < 0.01), DMT-Tepezcohuite (10.4%, χ^2^ = 38.43, *p* < 0.01), Ketamine (8.4%, χ^2^ = 35.70, *p* < 0.01), Synthetic DMT (8%, χ^2^ = 36.93, *p* < 0.01), and Ibogaine (1.4%, χ^2^ = 38.43, *p* < 0.01). Consuming in the company of a facilitator was associated with Ayahuasca (29.1%, χ^2^ = 172.76, *p* < 0.01), Psilocybin mushrooms (16.2%, χ^2^ = 34.34, *p* < 0.01), Peyote (13%, χ^2^ = 71.08, *p* < 0.01), San Pedro cactus (8.2%, χ^2^ = 95.58, *p* < 0.01), DMT-Tepezcohuite (2.4%, χ^2^ = 60.01, *p* < 0.01), and Anadenanthera Colubrina (1.5%, χ^2^ = 39.90, *p* < 0.01). Consuming with strangers was associated only with 2 C-B (3.5%, χ^2^ = 55.32, *p* < 0.01). Consuming in the company of a dealer was related to Ketamine (2.6%, χ^2^ = 63.59, *p* < 0.01), MDMA (2.6%, χ^2^ = 34.39, *p* < 0.01), and 2 C-B (2.3%, χ^2^ = 56.08, *p* < 0.01).

#### Acquisition

 Regarding the sources of acquisition of psychedelics, purchasing through a dealer was significantly associated with the regular use of Psilocybin mushrooms (χ^2^ = 36.83, *p* < 0.01), LSD (χ^2^ = 40.59, *p* < 0.01), Ketamine (χ^2^ = 37.86, *p* < 0.01), MDMA (χ^2^ = 38.95, *p* < 0.01), and 2 C-B (χ^2^ = 34.70, *p* < 0.01). Acquisition through a dealer via a mobile application was associated with Ketamine (χ^2^ = 42.38, *p* < 0.01) and 2 C-B (χ^2^ = 39.09, *p* < 0.01). Acquisition through friends was linked to the consumption of Ketamine (χ^2^ = 36.37, *p* < 0.01), MDMA (χ^2^ = 40.30, *p* < 0.01), and 2 C-B (χ^2^ = 36.37, *p* < 0.01). Acquisition through a facilitator was associated with the use of Peyote (χ^2^ = 63.94, *p* < 0.01), San Pedro cactus (χ^2^ = 81.09, *p* < 0.01), Ayahuasca (χ^2^ = 166.10, *p* < 0.01), Salvia Divinorum (χ^2^ = 35.10, *p* < 0.01), DMT-Tepezcohuite (χ^2^ = 60.51, *p* < 0.01), and Anadenanthera Colubrina (χ^2^ = 40.07, *p* < 0.01). Acquisition through a partner (χ^2^ = 38.11, *p* < 0.01) and/or a family member (χ^2^ = 35.26, *p* < 0.01) was associated with MDMA consumption. Home-growing was associated only with the use of San Pedro cactus (χ^2^ = 41.16, *p* < 0.01).

Considering that the use of psychedelics should account for the analysis of extra-pharmacological variables, described as “set and setting”^22–26^, we explored the relationship between the consumption of each substance and variables associated with consumption. We observed a significant relationship between ceremonial context, the company of a facilitator, and the consumption of Ayahuasca (χ^2^ = 252.36, *p* < 0.01), San Pedro cactus (χ^2^ = 251.88, *p* < 0.01), and Peyote (χ^2^ = 248.81, *p* < 0.01). Additionally, significant relationships were found between consumption in the company of friends at parties and the use of MDMA (χ^2^ = 56.95, *p* < 0.01), 2 C-B (χ^2^ = 57.69, *p* < 0.01), Ketamine (χ^2^ = 52.98, *p* < 0.01), and LSD (χ^2^ = 56.85, *p* < 0.01).

### Regular psychedelics use and perceived changes

 Significant relationships were observed between the positive perceived changes and the specific psychedelic substances regularly consumed (Table 5), as well as between negative perceived changes and the substances regularly consumed (Table 6).

**Table 5:** Positive changes reported and psychedelics used on a regular basis.

Positive changes reported	Psychedelics
Increased capacity of self-observation	Psilocybin mushrooms (66.4%, χ^2^ = 70.79, *p* < 0.01) LSD (36.1%, χ^2^ = 39.11, *p* < 0.01) MDMA (30%, χ^2^ = 35.97, *p* < 0.01) San Pedro cactus (14.1%, χ^2^ = 42.44, *p* < 0.01) Synthetic DMT (12.4%, χ^2^ = 45.35, *p* < 0.01) Ayahuasca (10.5%, χ^2^ = 39.62, *p* < 0.01) Floripondium (3%, χ^2^ = 34.19, *p* < 0.01)
Improved mood	Psilocybin mushrooms (57.4%, χ^2^ = 43.26, *p* < 0.01) San Pedro cactus (19.8%, χ^2^ = 35.70, *p* < 0.01)
Personal growth	Psilocybin mushrooms (54.1%, χ^2^ = 67.12, *p* < 0.01) LSD (53.8%, χ^2^ = 36.60, *p* < 0.01) San Pedro cactus (12.4%, χ^2^ = 43.54, *p* < 0.01) Ayahuasca (10.1%, χ^2^ = 47.43, *p* < 0.01) Synthetic DMT (9.6%, χ^2^ = 35.24, *p* < 0.01) Floripondium (2.9%, χ^2^ = 35.27, *p* < 0.01)
Heightened sensitivity	Psilocybin mushrooms (52.9%, χ^2^ = 61.75, *p* < 0.01) Ayahuasca (18.3%, χ^2^ = 36.39, *p* < 0.01) DMT-Tepezcohuite (13.2%, χ^2^ = 35.01, *p* < 0.01) San Pedro cactus (12.9%, χ^2^ = 42.83, *p* < 0.01) Synthetic DMT (9.2%, χ^2^ = 37.42, *p* < 0.01)
Spiritual growth	Psilocybin mushrooms (51.1%, χ^2^ = 55.71, *p* < 0.01) Ayahuasca (19.4%, χ^2^ = 53.52, *p* < 0.01) San Pedro cactus (13.1%, χ^2^ = 54.22, *p* < 0.01) Synthetic DMT (10.1%, χ^2^ = 40.69, *p* < 0.01) DMT-Tepezcohuite (9.3%, χ^2^ = 35.36, *p* < 0.01) Peyote (8.7%, χ^2^ = 39.63, *p* < 0.01) Floripondium (2.8%, χ^2^ = 36.16, *p* < 0.01)
Decrease in anxiety and/or distress	Psilocybin mushrooms (49.9%, χ^2^ = 40.65, *p* < 0.01) DMT-Tepezcohuite (6.2%, χ^2^ = 35.31, *p* < 0.01)
Positive changes in my eating, hygiene and/or self-care habits	Psilocybin mushrooms (47.5%, χ^2^ = 43.66, *p* < 0.01)
Increased questioning of my work, relationships and/or habits, without discomfort	Psilocybin mushrooms (43%, χ^2^ = 40.41, *p* < 0.01) LSD (19.9%, χ^2^ = 35.57, *p* < 0.01) MDMA (17.3%, χ^2^ = 36.92, *p* < 0.01) Synthetic DMT (8.4%, χ^2^ = 44.49, *p* < 0.01) San Pedro cactus (7.7%, χ^2^ = 34.86, *p* < 0.01)
Decreased fear of dying and/or of losing something/someone	Ayahuasca (44%, χ^2^ = 42.43, *p* < 0.01) Psilocybin mushrooms (22%, χ^2^ = 39.66, *p* < 0.01) Synthetic DMT (15.2%, χ^2^ = 42.31, *p* < 0.01) LSD (12.8%, χ^2^ = 35.13, *p* < 0.01) DMT-Tepezcohuite (5.4%, χ^2^ = 43.74, *p* < 0.01) San Pedro cactus (5.2%, χ^2^ = 34.83, *p* < 0.01) Peyote (3.3%, χ^2^ = 42.85, *p* < 0.01)
Increased problem-solving skills	Psilocybin mushrooms (39.1%, χ^2^ = 52.65, *p* < 0.01) San Pedro cactus (22.8%, χ^2^ = 38.56, *p* < 0.01) DMT-Tepezcohuite (12%, χ^2^ = 39.11, *p* < 0.01) Ayahuasca (7.4%, χ^2^ = 36.09, *p* < 0.01) Synthetic DMT (8%, χ^2^ = 38.81, *p* < 0.01)
Changes in my recreational interests	Psilocybin mushrooms (38.6%, χ^2^ = 41.65, *p* < 0.01) Ayahuasca (29%, χ^2^ = 37.88, *p* < 0.01) San Pedro cactus (18.8%, χ^2^ = 35.71, *p* < 0.01) LSD (16.6%, χ^2^ = 34.70, *p* < 0.01) MDMA (15.5%, χ^2^ = 40.87, *p* < 0.01) Synthetic DMT (12.6%, χ^2^ = 34.10, *p* < 0.01) DMT-Tepezcohuite (12%, χ^2^ = 38.47, *p* < 0.01) Peyote (3%, χ^2^ = 46.48, *p* < 0.01)
General reduction of fears	Psilocybin mushrooms (36.4%, χ^2^ = 57.68, *p* < 0.01) LSD (20.6%, χ^2^ = 36.56, *p* < 0.01) Ayahuasca (11.6%, χ^2^ = 37.65, *p* < 0.01) Synthetic DMT (7.9%, χ^2^ = 34.70, *p* < 0.01) DMT-Tepezcohuite (5%, χ^2^ = 40.57, *p* < 0.01) Floripondium (2.1%, χ^2^ = 34.80, *p* < 0.01)
Distancing from people with whom I used to share frequently, without discomfort	Psilocybin mushrooms (22.3%, χ^2^ = 44.51, *p* < 0.01) Ayahuasca (24%, χ^2^ = 46.07, *p* < 0.01) San Pedro cactus (16.3%, χ^2^ = 42.43, *p* < 0.01) Peyote (10.8%, χ^2^ = 31.79, *p* < 0.01) DMT-Tepezcohuite (9.9%, χ^2^ = 50.98, *p* < 0.01) Synthetic DMT (4.7%, χ^2^ = 35.11, *p* < 0.01)
Increased creativity	San Pedro cactus (14.5%, χ^2^ = 44.62, *p* < 0.01) Peyote (5.3%, χ^2^ = 42.09, *p* < 0.01)
Physical well-being	San Pedro cactus (8.7%, χ^2^ = 43.33, *p* < 0.01) Peyote (8.2%, χ^2^ = 38.96, *p* < 0.01) Ayahuasca (7.9%, χ^2^ = 42.56, *p* < 0.01) Synthetic DMT (6.6%, χ^2^ = 35.56, *p* < 0.01) DMT-Tepezcohuite (3.3%, χ^2^ = 41.83, *p* < 0.01) Floripondium (2.1%, χ^2^ = 35.93, *p* < 0.01)

All *X*^2^ significance test contrasts were performed at the *p* < 0.01 and corrected using the FDR method.

**Table 6:** Negative changes reported and psychedelics used on a regular basis.

Negative changes reported	Psychedelics
Decreased memory capacity	2 C-B (49.8%, χ^2^ = 71.86, *p* < 0.01) Ketamine (34.4%, χ^2^ = 61.08, *p* < 0.01)
Negative changes in my eating, hygiene and/or self-care habits	2 C-B (46.7%, χ^2^ = 41.91, *p* < 0.01) Ketamine (24.9%, χ^2^ = 37.39, *p* < 0.01)
Some degree of discomfort when sharing with groups of people (known or unknown)	2 C-B (43.2%, χ^2^ = 23.04, *p* < 0.01) Synthetic DMT (3.7%, χ^2^ = 24.17, *p* < 0.01) San Pedro cactus (2.9%, χ^2^ = 20.30, *p* < 0.01)
Worse mood	2 C-B (41.7%, χ^2^ = 36.36, *p* < 0.01)
Increased difficulty in concentrating on work and/or day-to-day activities	2 C-B (35.1%, χ^2^ = 42.44, *p* < 0.01)
Physical discomfort	Ketamine (29.7%, χ^2^ = 39.24, *p* < 0.01) 2 C-B (18.4%, χ^2^ = 49.51, *p* < 0.01)
Increased questioning of my work, relationships and/or habits, with discomfort	2 C-B (24.4%, χ^2^ = 39.33, *p* < 0.01)

All *X*^2^ significance test contrasts were performed at the *p* < 0.01 and corrected using the FDR method.

Additionally, “changes in my recreational interests” were related to both female gender (χ^2^ = 36.06, *p* < 0.01) and male gender (χ^2^ = 35.83, *p* < 0.01).

## Discussion

The present study aimed to characterize the variables associated with the regular use of macrodoses of psychedelics in the Latin American context.

Our findings on the age of first consumption differ from previous studies^27,28^, which suggest that the onset of substance use typically occurs between 14 and 18 years of age. According to our data, the first consumption experience happens around the age of 24 years. This suggests that within our sample, individuals may make consumption decisions with greater maturity and awareness, possibly supported by a broader knowledge of the substances involved. Although psychedelics are considered drugs, their use seems to follow a different onset pattern than substances such as cocaine and opiates.

Previous studies^29^ link the use of substances such as MDMA and Ketamine to the 2SLGBTQ + community; however, our findings show no relationship between sexual orientation and psychedelic use within our sample. Regarding gender, earlier reports^30^ indicate that LSD use is higher in men than in women. In our sample, we observed that LSD and Ketamine use is associated with both women and men, while DMT use is only associated with men. The non-binary gender did not show any association with the consumption of any substance.

Our results indicate that 92.8% of the participants have technical and/or university education, and 93.6% are either working or studying. This finding contrasts with one of the most common misconceptions in the general community, which assumes that psychedelic users are people with low educational levels and limited job potential^31^. Additionally, our findings show specific relationships between occupation and regularly used substances within our sample. We observed that students are associated with the consumption of Psilocybin mushrooms and LSD, while being a facilitator of psychedelic experiences is associated with the consumption of Ayahuasca, DMT, and DMT-Tepezcohuite. Only the use of Psilocybin mushrooms was associated with 13 or more years of education.

Regarding socioeconomic status, previous findings have associated substance use with low family socioeconomic levels^32^. In our sample, the monthly income of participants is related to the type of substance consumed. For example, monthly incomes up to USD $698 are associated with the consumption of Psilocybin mushrooms and 2 C-B, while the consumption of substances such as MDMA and Ketamine is associated with monthly incomes above USD $3,584. This suggests that in our sample, socioeconomic level is more related to the type of substance people have access to, rather than to consumption itself.

In relation to religion, previous evidence shows a connection between religiosity/spirituality and substance use, with higher levels of religiosity/spirituality generally associated with lower substance use^33^. Our results partially align with this evidence. On the one hand, most participants do not belong to any formal religion, which is associated with the consumption of Psilocybin mushrooms, Ayahuasca, Salvia Divinorum, and 2 C-B. On the other hand, people who profess a shamanic creed are associated with the consumption of Ayahuasca, Peyote, and San Pedro cactus. This is logical considering the substances involved, the context of consumption (ceremony or retreat), and their connection with the practices of Native American cultures, where these substances are considered sacred medicines and their consumption is part of their cosmovisión.

Global reports on the prevalence of substance use vary greatly. The 2023 World Drug Report mentions that in Australia and New Zealand, MDMA is the most prevalent^1^, while in the USA, 9.68% of the population consumes Psilocybin mushrooms^34^. Our results are consistent with these reports, indicating that in the Latin American context, Psilocybin mushrooms are the most consumed substance, followed by MDMA within our sample. It is important to note that these findings do not consider aspects related to the supply and demand of these psychedelics in the illegal market, representing only indicators of the substances that people prefer to consume within our sample.

Previous studies have explored the motivations associated with psychedelic use, identifying several key drivers. The primary motives include the desire to expand (self-)knowledge and curiosity^35^, promote spiritual development^35,39^, access altered states of consciousness, enhance sexual activity, engage in psychotherapy, boost creativity^39^ and/or connect with nature^39,37^. Additionally, a qualitative report on a small sample of regular psychedelic users describes “fun” and “existential quest” as the main motivations^41^. Furthermore, it has been reported that mescaline use may be driven by spiritual exploration^37^. Our results suggest that the motivations for macrodosing within our sample are also mainly related to the recreational experience, psychological well-being and spiritual growth experienced after consumption. Additionally, we observed that consumption for recreational purposes is the most frequent within our sample; however, consumption for spiritual purposes also represents a very high percentage. A significant percentage of participants describe more than one purpose for regular consumption, and the statistics show a significant relationship between these two purposes.

Similar to previous findings^27^, our results show that most participants prefer to consume psychedelics in the company of others. It can be hypothesized that given the complexity of the psychological experience induced by psychedelics, the company of others provides security, containment, and support. On the other hand, we also observed that 49.2% of the participants practice solitary consumption.

The results show significant relationships between certain contexts of consumption and the substances consumed, highlighting the importance of considering variables associated with the setting. Previous reports describe a preference for ceremonial contexts^27^, while our findings describe a preference for consumption at home or outdoors within our sample. Additionally, we observed significant relationships between the substance consumed, the context of consumption, and the company. For example, consuming Ayahuasca, San Pedro cactus, and Peyote is related to ceremonial contexts and the company of a facilitator, while the use of Ketamine, MDMA, LSD, and 2 C-B is related to parties and being with friends.

North American users have been reported to use psychedelics frequently^39^. In the absence of a clear representation of frequency of use, our results show that most participants in our sample prefer to use psychedelics only a few times a year. The difficulty in uniquely operationalizing the concept of frequency may be related to the differences observed between studies.

There are no known global reports detailing the specific sources of psychedelic acquisition. Our results indicate that most participants in our sample obtain these substances through a dealer or a third party. This is understandable in the Latin American context, where the adult and responsible use of psychedelics is prohibited by the legal framework in most countries. The findings suggest that these prohibitive laws do not prevent consumption but instead encourage illicit trafficking and the potential social and health consequences of illegal use, thereby increasing health risks for the population^40^.

Regarding the subjective changes associated with the regular use of psychedelics, it is notable that those considered negative are less frequent than those considered positive. Additionally, the negative perception of subjective effects is mainly associated with the regular use of synthetic psychedelics, specifically 2 C-B and Ketamine. On the other hand, positive effects are primarily associated with psychedelics of organic origin. Among the relationships described, Psilocybin mushrooms showed the most significant relationships between use and experienced well-being within our sample.

Our study has several important strengths, including a large sample size and the inclusion of psychedelic users from various contexts of consumption within Latin America. However, several limitations should be noted. The use of a non-probabilistic, self-selected sample introduces bias towards participants who have had positive experiences and are motivated to complete a survey, potentially limiting the generalizability of the findings to the wider population^19,27^ of psychedelic users in Latin America. Additionally, the data collection instrument relied entirely on self-report, which can introduce biases such as recall bias, especially since several items involved retrospective assessments^19^. Despite the inclusion of attentional control questions to enhance data quality, self-report data inherently carries the risk of inaccuracies^41^. The cross-sectional design of the study does not provide data on the mental health status of respondents prior to their psychedelic use, which means that individuals with better mental health may be more likely to report improved psychological well-being, reflecting their better past mental health status^19^. Furthermore, we lack detailed information on the composition, purity, and dose (concentration and quantity) of the psychedelics consumed, which could influence the outcomes, as variability in these factors may affect the reported experiences and associations observed^19^. While specific practices may influence the experiences and mental health outcomes of psychedelic users, other aspects of the ceremony and additional support may be equally or more important; our data regarding the context were limited to the motivations of users, without considering other factors like the mental state at the time of ingestion^19^. The recruitment method, utilizing web-based announcements on social media services, advertisements, and interviews, may not capture older generations and/or psychedelic users who do not use the Internet or social media platforms, thus missing older users whose experiences could significantly differ from current trends^27^. Finally, the study’s quantitative focus offers a somewhat limited view of the complex phenomenon of psychedelic use, and to better understand such a multifaceted issue, longitudinal research incorporating both quantitative and qualitative designs would be beneficial^27^. These limitations suggest caution when interpreting our findings and highlight the need for future research to address these issues. Future studies should aim to incorporate more diverse sampling methods, detailed information on the substances used, and a combination of quantitative and qualitative approaches to provide a comprehensive understanding of psychedelic use and its effects.

Considering the study’s design, the relationships presented here are exploratory in nature. Therefore, we believe these findings provide a foundation for future research that delves deeper into these and other interactions, focusing on moderation, mediation, or explanatory models of these relationships.

Future research should prioritize longitudinal studies to understand the long-term effects of regular psychedelic use and conduct comparative studies between different regions to uncover cultural and socioeconomic influences. Detailed analyses of set and setting in psychedelic experiences, particularly in naturalistic settings, could offer valuable insights into their therapeutic potential. Additionally, examining microdosing practices alongside macrodosing could provide a comprehensive understanding of the impacts of different dosing strategies. Developing harm reduction strategies based on these findings will be crucial for ensuring safe consumption. Finally, investigating the impact of regular psychedelic use on community and social dynamics through interdisciplinary and ethnographic studies will deepen our understanding of its broader social implications.

The production of this knowledge would help generate an informed, evidence-based dialogue, fostering a paradigm shift that lays the groundwork for the informed, safe, and legal use of these substances. It would also contribute to breaking down stigmas, misconceptions, and prejudices associated with these practices, paving the way for new research in the Latin American context. This information is relevant for Latin American governments as they consider reviewing current regulatory frameworks on psychedelic use, taking into account the variables associated with consumption and the promising therapeutic potential of these substances.

## Electronic supplementary material

Below is the link to the electronic supplementary material.

Supplementary Material 1

## Data Availability

Data is available upon reasonable request to the corresponding author.

## References

[1] UNODC, Informe mundial sobre las drogas 2023 publicación de las Naciones Unidas (2023).

[2] Carod-Artal, F. J. Hallucinogenic drugs in pre-columbian mesoamerican cultures. *Neurologia* **30**(1), 42–49. 10.1016/j.nrleng.2011.07.010 (2015). 21893367 10.1016/j.nrl.2011.07.003

[3] McBride, M. Allopathic shamanism: Indigenous American cultures, psychopharmacy, and the prince of flowers. In *Breathe and Smoke* 93–125 (University of New Mexico Press, 2019).

[4] Nichols, D. *Psychedelics: Phenomenology and brain chemistry* 266–268 (Lunaria Editions, 2019).

[5] Timmermann, C. Neurociencias Y aplicaciones psicoterapéuticas en El renacimiento de la investigación con psicodélicos. *Revista Chil. De neuro-psiquiatría* **52**(2), 93–102. 10.4067/S0717-92272014000200005 (2014).

[6] Gómez-Escolar, A. *Guía Esencial del Renacimiento Psicodélico.* 80–87Alpha Editorial, (2022).

[7] Hadar, A. et al. The psychedelic Renaissance in Clinical Research: A bibliometric analysis of three decades of Human studies with Psychedelics. *J. Psychoact Drugs* **55**(1). 10.1080/02791072.2021.2022254 (2023). 10.1080/02791072.2021.2022254 35000572

[8] Nutt, D. & Carhart-Harris, R. The current status of psychedelics in psychiatry. *JAMA Psychiatry* **78**(2). 10.1001/jamapsychiatry.2020.2171 (2021). 10.1001/jamapsychiatry.2020.2171 32725172

[9] Ross, S. et al. Rapid and Sustained Symptom Reduction following psilocybin treatment for anxiety and depression in patients with life-threatening Cancer: A Randomized Controlled Trial. *J. Psychopharmacol.* **30**(12), 11651180. 10.1177/0269881116675512 (2016). 10.1177/0269881116675512 PMC5367551 27909164

[10] Grob, C. S. et al. Pilot study of psilocybin treatment for anxiety in patients with advanced-stage cancer. *Arch. Gen. Psychiatry* **68**(1). 10.1001/archgenpsychiatry.2010.116 (2011). 10.1001/archgenpsychiatry.2010.116 20819978

[11] Griffiths, R. R. et al. Psilocybin occasioned mystical-type experiences: Immediate and persisting dose-related effects. *Psychopharmacology* **218**(4). 10.1007/s00213-011-2358-5 (2011). 10.1007/s00213-011-2358-5 PMC3308357 21674151

[12] Ragnhildstveit, A. et al. 5-MeO-DMT for post-traumatic stress disorder: A real-world longitudinal case study. *Front. Psychiatry* **14**10.3389/fpsyt.2023.1271152 (2023). 10.3389/fpsyt.2023.1271152 PMC10710141 38076677

[13] Oehen, P., Traber, R., Widmer, V. & Schnyder, U. A randomized, controlled pilot study of MDMA (+/– 3,4-Methylenedioxymethamphetamine)-assisted psychotherapy for treatment of resistant, chronic post-traumatic stress disorder (PTSD). *J. Psychopharmacol.* **27**, 40–52. 10.1177/0269881112464827 (2013). 23118021 10.1177/0269881112464827

[14] Mithoefer, M. C. et al. 3,4-methylenedioxymethamphetamine (MDMA)-assisted psychotherapy for post-traumatic stress disorder in military veterans, firefighters, and police officers: A randomised, double-blind, dose-response, phase 2 clinical trial. *Lancet Psychiatry* **5**, 486–497. 10.1016/S2215-0366(18)30135-4 (2018). 29728331 10.1016/S2215-0366(18)30135-4

[15] Ot’alora, G. M. et al. 3,4-Methylenedioxymethamphetamine-assisted psychotherapy for treatment of chronic posttraumatic stress disorder: A randomized phase 2 controlled trial. *J. Psychopharmacol.* **32**, 1295–1307. 10.1177/0269881118806297 (2018). 30371148 10.1177/0269881118806297 PMC6247454

[16] Bogenschutz, M. et al. Psilocybin-assisted treatment for Alcohol Dependence: A Proof-Of-Concept Study. *J. Psychopharmacol.* **29**(3). 10.1177/0269881114565144 (2015). 10.1177/0269881114565144 25586396

[17] Mash, D. C., Duque, L., Page, B. & Allen-Ferdinand, K. Ibogaine detoxification transitions opioid and cocaine abusers between dependence and abstinence: Clinical observations and treatment outcomes. *Front. Pharmacol.*** 9** (2018). 10.3389/fphar.2018.00529 10.3389/fphar.2018.00529 PMC5996271 29922156

[18] Johnstad, P. G. Who is the typical psychedelics user? Methodological challenges for research in psychedelics use and its consequences. *Nordic Stud. Alcohol Drugs* **38**(1). 10.1177/1455072520963787 (2021). 10.1177/1455072520963787 PMC8899058 35309094

[19] Perkins, D. et al. Influence of context and setting on the mental health and wellbeing outcomes of ayahuasca drinkers: Results of a large International Survey. *Front. Pharmacol.* **12**, 623979. 10.3389/fphar.2021.623979 (2021). 33967757 10.3389/fphar.2021.623979 PMC8097729

[20] Irwin, A. *Citizen Science. 2–4* (London Routledge, 1995).

[21] Benjamini, Y. & Hochberg, Y. Controlling the false discovery rate: A practical and powerful approach to multiple testing. *J. Roy. Stat. Soc.: Ser. B (Methodol.)* **57**(1). 10.1111/j.2517-6161.1995.tb02031.x (1995).

[22] Hartogsohn, I. *The psycho-social construction of LSD: How set and setting shaped the American psychedelic experience 1950–1970* (Bar Ilan University, 2015).

[23] Dwyer, R. & Moore, D. Enacting multiple methamphetamines: The ontological politics of public discourse and consumer accounts of a drug and its effects. *Int. J. Drug Policy* **24**(3). 10.1016/j.drugpo.2013.03.003 (2013). 10.1016/j.drugpo.2013.03.003 23540297

[24] Hart, C. *High Price: A Neuroscientist’s Journey of Self-Discovery that Challenges Everything You Know About Drugs and Society* (Harper Collins, 2013).

[25] Zinberg, N. E. *Drug, set, and Setting: The Basis for Controlled Intoxicant use* 1st edn (Yale University Press, 1984).

[26] Leary, T., Litwin, G. H. & Metzner, R. Reactions to psilocybin administered in a supportive environment. *J. Nervous Mental Dis.* **137**. 10.1097/00005053-196312000-00007 (1963). 10.1097/00005053-196312000-00007 14087676

[27] Lukačovič, M. & Masaryk, R. Use of hallucinogens in Slovakia: Does it differ from global trends? *Int. J. Drug Policy* **98**. 10.1016/j.drugpo.2021.103385 (2021). 10.1016/j.drugpo.2021.103385 34364200

[28] Ruiz, H., Herrera, A., Martínez, A. & y Supervielle, M. Comportamiento Adictivo De La familia como factor de riesgo de consumo de drogas en jóvenes y adolescentes adictos. *Revista Cubana Invest. Bioméd* **33**(4) http://scielo.sld.cu/scielo.php?script=sci_arttext (2014).

[29] Dharma, C. et al. Factors associated with the use of psychedelics, ketamine and MDMA among sexual and gender minority youths in Canada: A machine learning analysis. *J. Epidemiol. Community Health* **78**10.1136/jech-2023-220748 (2024). 10.1136/jech-2023-220748 38262735

[30] Krebs, T. S. & Johansen, P. Ø. Over 30 million psychedelic users in the United States. *F1000Research* **2** (2013). 10.12688/f1000research.2-98.v1 10.12688/f1000research.2-98.v1 PMC3917651 24627778

[31] Nieweglowski, K. et al. Exploring the public stigma of substance use disorder through community-based participatory research. *Addict. Res. Theory* **26**(4). 10.1080/16066359.2017.1409890 (2018).

[32] Aschengrau, A. et al. Influence of family and community socioeconomic status on the risk of adolescent drug use. *Subst. Use Misuse* **56**10.1080/10826084.2021.1883660 (2021). 10.1080/10826084.2021.1883660 33719860

[33] Acheampong, A. B. et al. Gender differences in the association between religion/spirituality and simultaneous polysubstance use (SPU). *J. Relig. Health* **55**(5). 10.1007/s10943-015-0168-5 (2015). 10.1007/s10943-015-0168-5 PMC8341450 26693722

[34] Yockey, A. & King, K. Use of psilocybin (mushrooms) among US adults: 2015–2018. *J. Psychedelic Stud.* **5**(1). 10.1556/2054.2020.00159 (2021).

[35] Basedow, L. A. & Kuitunen-Paul, S. Motives for the use of serotonergic psychedelics: A systematic review. *Drug Alcohol Rev.* **41**(6), 1391–1403 10.1111/dar.13480 (2022). 10.1111/dar.13480 35668698

[36] Roberts, C. A., Osborne-Miller, I., Cole, J., Gage, S. H. & Christiansen, P. Perceived harm, motivations for use and subjective experiences of recreational psychedelic ‘magic’mushroom use. *J. Psychopharmacol.* **34**(9), 999–1007. 10.1177/0269881120936508 (2020). 32674668 10.1177/0269881120936508

[37] Uthaug, M. V. et al. The epidemiology of mescaline use: Pattern of use, motivations for consumption, and perceived consequences, benefits, and acute and enduring subjective effects. *J. Psychopharmacol.* **36**(3), 309–320. 10.1177/02698811211013583 (2022). 10.1177/02698811211013583 PMC8902264 33949246

[38] Dollar, C. B. Recreation and realization: Reported motivations of use among persons who consume psychedelics in non-clinical settings. *J. Qual. Crim. Justice Criminol.* **10**(4), 1–41. 10.21428/88de04a1.cb6fef95 (2021).

[39] Kruger, D. J. et al. An Assessment of psychedelic knowledge among people using psychedelics naturalistically. *J. Psychoactive Drugs* **55**(4). 10.1080/02791072.2022.2142709 (2023). 10.1080/02791072.2022.2142709 36328419

[40] Hughes, C. E. & Stevens, A. What can we learn from the Portuguese decriminalization of Illicit drugs. *Br. J. Criminol.* **50**, 999–1022. 10.1093/bjc/azq038 (2010).

[41] Domic-Siede, M. et al. Emotion regulation unveiled through the categorical lens of attachment. *BMC Psychol.* **12**(1), 240. 10.1186/s40359-024-01748-z (2024). 38678214 10.1186/s40359-024-01748-z PMC11056069

