# Supplementary material for: Latin American adults who regularly use macrodoses of psychedelics: a cross-sectional study
Source: Sci Rep. 2024 Oct 13;14:23921. doi: 10.1038/s41598-024-74590-3 (PMC11471782; doi:10.1038/s41598-024-74590-3)
Supplement: Supplementary file 1 — Supplementary Material 1 [file 41598_2024_74590_MOESM1_ESM.docx]

Supplementary Table A. Specification of each psychedelic considered in the study.

| **Psychedelic Substance** | **Other names (street names and/or native names)** | **Psychoactive compounds** |
| --- | --- | --- |
| **Psilocybin mushrooms**  Hypholoma caerulescens, Hypholoma coerulescens, Naematoloma caerulescens, Nematoloma caerulescens, Psilocybe cubensis var. Caerulescens, Psilocybe cubensis var. Cubensis, Psilocybe cubensis var. cyanescens, Stropharia cubensis, and Stropharia cyanescens | Magic Mushrooms, Hallucinogenic Mushrooms, Mushies, Shrooms, Teonanacatl, Mexican Hats, Cucumelo | Psilocybin, Psilocin, Baeoicistin, Alkaloids |
| **Ayahuasca**  Preparation composed of Banisteriopsis Caapi, and Psychotria Viridis (Chakruna) | Grandmother, Yagé, Oní, Uní, Daime | N,N-dimethyltryptamine, Harmine, Tetrahydroharmine |
| **San Pedro cactus**  Echinopsis Pachanoi | Grandfather, Achuma, Aguacolla, Huachuma | Mescaline |
| **Peyote**  Lophophora Williamsii | Jícuri, Mescal Button, Challote, Mescalito | Mescaline |
| **Floripondium**  Brugmansia Arborea | Angel's Trumpets, Bell, Huacachaca, Bijaura, Queen of the Night, Brugmansia | Scopolamine, Atropine, Hyoscyamine |
| **Ibogaine**  Taberntanthe Iboga, Voacanga Iboga, Tabernaemontana, Tabernanthe Manii | Iboga, Eboka, Kwaho, Root of Wisdom | Ibogaine |
| **Salvia Divinorum** | Ska María Pastora, Grass of the Gods, Sage of the Diviners, Stone of Madness, Sage of Dreams, Pasangán | Salvinorin A |
| **Anandenanthera Colubrina**  Piptadenia Macrocarpa var. Cebil | Cebil, Vilca, Yopo, Cohoba, Curupay, Jataj | Bufotenin |
| **Tepezcohuite**  Smokable preparation combining some or all these plants: Mimosa Tenuiflora, Peganum Harmala, Banisteriopsis Caapi, Passiflora Incarnata, Acacia Maidenii, Acacia Obtusifolia, and Acacia Acuminata | Xanga, Changa, Tepez, Jurema, DMT-Tepezcohuite | N,N-dimethyltryptamine, Indole Alkaloids, Harmaline |
| **MDMA**  3,4- methylenedioxymethamphetamine | Ecstasy, Pile, Pasti, Molly, XTC | 3,4- methylenedioxymethamphetamine |
| **LSD**  Lysergic Acid Diethylamide | Acid, Stamp, Mellow Yellow, Tripy, Blotter Acid, Dots, Acid Gummies | Lysergic Acid Diethylamide |
| **Synthetic DMT**  N,N-dimethyltryptamine | Dimitri, Stardust, Crystal, Fantasy | N,N-dimethyltryptamine |
| **2C-B**  4-bromine-2,5- dimethoxyphenylethylamine | Nexus, Tucibí, Tusi, Pink Panther, Pink Cocaine, Bromo-mescaline | 4-bromine-2,5- dimethoxyphenylethylamine |
| **Ketamine**  (±)-2-(2-clorofenil)-2-(metilamino)ciclohexanona | K, Vitamin K, Special K, Cat Valium, Ket, Keta, Kit Kat, Green K, Honey Oil, Jet, Bump, Color, Flote | Ketamine |
